# Influence of rub-in technique on required application time and hand coverage in hygienic hand disinfection

**DOI:** 10.1186/1471-2334-8-149

**Published:** 2008-10-29

**Authors:** Günter Kampf, Mirja Reichel, Yvonne Feil, Sven Eggerstedt, Paul-Michael Kaulfers

**Affiliations:** 1 BODE Chemie GmbH & Co. KG, Scientific Affairs, Melanchthonstr. 27, 22525 Hamburg, Germany; 2 Institut für Hygiene und Umweltmedizin, Ernst-Moritz-Arndt Universität Greifswald, Walther-Rathenau-Str. 49a, 17475 Greifswald, Germany; 3 Institut für Pharmazie, Universität Hamburg, Bundesstr. 45, 20146 Hamburg, Germany; 4 BODE Chemie GmbH & Co. KG, Development, Melanchthonstr. 27, 22525 Hamburg, Germany; 5 Institut für Medizinische Mikrobiologie, Virologie und Hygiene, Universitätsklinikum Hamburg, Gebäude O26, Martinistr. 52, 20246 Hamburg, Germany

## Abstract

**Background:**

Recent data indicate that full efficacy of a hand rub preparation for hygienic hand disinfection can be achieved within 15 seconds (s). However, the efficacy test used for the European Norm (EN) 1500 samples only the fingertips. Therefore, we investigated hand coverage using sixteen different application variations. The hand rub was supplemented with a fluorescent dye, and hands were assessed under UV light by a blind test, before and after application. Fifteen non-healthcare workers were used as subjects for each application variation apart from one test which was done with a group of twenty healthcare workers. All tests apart from the reference procedure were performed using 3 mL of hand rub. The EN 1500 reference procedure, which consists of 6 specific rub-in steps performed twice with an aliquot of 3 ml each time, served as a control. In one part of this study, each of the six steps was performed from one to five times before proceeding to the next step. In another part of the study, the entire sequence of six steps was performed from one to five times. Finally, all subjects were instructed to cover both hands completely, irrespective of any specific steps ("responsible application"). Each rub-in technique was evaluated for untreated skin areas.

**Results:**

The reference procedure lasted on average 75 s and resulted in 53% of subjects with at least one untreated area on the hands. Five repetitions of the rub-in steps lasted on average 37 s with 67% of subjects having incompletely treated hands. One repetition lasted on average 17 s, and all subjects had at least one untreated area. Repeating the sequence of steps lasted longer, but did not yield a better result. "Responsible application" was quite fast, lasting 25 s among non-healthcare worker subjects and 28 s among healthcare workers. It was also effective, with 53% and 55% of hands being incompletely treated. New techniques were as fast and effective as "responsible application". Large untreated areas were found only with short applications. Fingertips and palms were often covered completely.

**Conclusion:**

In clinical practice, hand disinfection is apparently better than practitioners of infection control often anticipate. Based on our data, a high-quality hygienic hand disinfection is not possible within 15 s. A 30 s application time can, however, be recommended for clinical practice. The currently recommended six steps of EN 1500 are not really suitable for clinical practice, because they yield comparably poor results. The most appropriate application procedure may be "responsible application", or one of the other new techniques.

## Background

In many European countries the commonly recommended application time for hygienic hand disinfection is 30 seconds (s), based on efficacy data obtained according to EN 1500 [1]. Well-formulated hand rubs, however, may show *in vitro *bactericidal activity in only 15 seconds [2]. Therefore, a sufficient efficacy according to EN 1500 might also be achieved *in vivo *in an application time shorter than 30 seconds. One major concern, however, is that a 15 s treatment would not ensure that the hands are entirely covered with the hand disinfectant. The Centers for Disease Control and Prevention (CDC) guidelines for hand rub use recommend a very general technique when using a hand rub: the product should be applied to the palm of one hand, and the hands should be rubbed together, covering all surfaces of hands and fingers, until the hands are dry [3]. A similar recommendation is provided in the hand hygiene guideline published by the Robert Koch Institute [4].

In 1978 Taylor looked at the duration and effectiveness of hand washing using a dye in alcohol [5]. A total of 129 procedures were observed. The median duration of the hand washing was 20 or 21 s [5,6]. Eighty-nine percent of the procedures missed some parts of the hands, indicating that the individual hand washing technique of the healthcare worker subjects was poor [5]. Based on this observation, Ayliffe proposed a specific rub-in technique which is now the basis for the EN 1500 [7]. Only a few studies are available for alcohol-based hand rubs. In 1996, it was reported that among 150 healthcare professionals, 57% had untreated skin areas on the thumbs, and 35% had untreated skin areas on the finger tips [8]. In another survey, a large proportion of 60 infection control professionals failed to perform an appropriate hand rub technique [9]. Another study on 180 healthcare workers found that only 31% followed the recommended rub-in technique that is described in EN 1500 [10].

The original efficacy test for hygienic hand disinfection published in 1977 did not specify a rub-in technique at all [11]. Since 1997, a specific rub-in technique [7] based on the findings from Taylor [5] has been described in EN 1500 as the reference procedure that ensures a complete coverage of hands with the reference alcohol [12]. It consists of 2 consecutive treatments with 3 mL, each with 6 specific steps that should be repeated five times. A short application like 15 s may not be sufficient to cover both hands entirely, and different types of hand rubbing techniques, to our knowledge, have never been studied systematically. Therefore, we studied various rub-in techniques with the aim of determining the mean duration of each technique, and the proportion of completely treated hands given by each procedure.

## Methods

### Hand rub preparations

The reference disinfectant, 2-propanol (60%, v/v), was used as described in EN 1500 [12]. Three mL of the reference alcohol, supplemented with 0.98% of fluorescent dye (Visirub, Bode Chemie GmbH & Co. KG, Hamburg, Germany) was applied twice to the hands for a total of 6 mL. In all other applications, 3 mL of a propanol-based hand rub (Sterillium; Bode Chemie GmbH & Co. KG, Hamburg, Germany) supplemented with 1.96% of the same fluorescent dye was applied once to the hands for a total of 3 mL. Different concentrations of the fluorescent dye were chosen to ensure that the total amount of dye on the hands would be the same, irrespective of the type of treatment.

### Test subjects

Fifteen test subjects (office workers, laboratory technicians, chemists and purchasers), were recruited for each application variation. None were healthcare professionals. Most have never been trained before to perform a specific rub-in technique. Non-healthcare workers were chosen in order to have a group of volunteers who were not routinely involved in the application of alcohol-based hand rubs, and who would allow a better assessment of the practicability of the currently recommended six steps. In this part of the study, as soon as the hand disinfection procedure was finished, the test was stopped and time recorded.

Another part of the study was done in the Cardiology Center of the University Hospital of Hamburg, with twenty healthcare workers (nurses and doctors) participating. They were told to do whatever they considered necessary to completely cover both hands with 3 mL ("responsible application"). The time was stopped and recorded when the subject thought both hands were covered completely.

From all subjects, the following information was obtained: gender, age and dominant hand. 138 of the 245 subjects were female (56.6%); the mean age was 39.9 years (range: 19 – 58 years); and 78.0% of the subjects were right-handed. Ethical approval was not considered necessary to carry out the study.

### Variations of the rub-in technique

Immediately before the hand rub was applied to the hands, volunteers were trained on exactly how to perform the various steps of the hand rub procedure to ensure that the subject knew exactly what to do. All subjects were told to perform the application at their own speed, not to hurry and not to waste time. The standard mode of application of hand rubs is described in EN 1500 [12]. It consists of 6 steps:

Step 1: Palm to palm.

Step 2: Right palm over back of left hand and left palm over back of right hand.

Step 3: Palm to palm with fingers interlaced.

Step 4: Back of fingers to opposing palms with fingers interlocked.

Step 5: Rotational rubbing of right thumb clasped in left palm and vice versa.

Step 6: Rotational rubbing, backwards and forwards, with fingertips of right hand in left palm and vice versa.

Variation 1: The reference procedure was tested as described in EN 1500. Three mL of the reference alcohol were applied to the hands. Each step was repeated five times beginning with step 1. The whole procedure was repeated with a second aliquot of 3 mL.

Variations 2 – 6 (repeating each step before performing the next step): Three mL were applied once. Each step was performed one, two, three, four or five times before the next step was performed.

Variations 7 – 11 (repeating the sequence of steps): Three mL were applied once. The whole sequence of the six steps was performed one, two, three, four or five times.

Variation 12: Three mL were applied once. Subjects were told to use their own individual technique to make sure that the whole hand was covered ("responsible application"). No specific rub-in instructions were given.

Variation 13: Three mL were applied once. The twenty healthcare workers from the Cardiology Center of Hamburg were told to make sure that the whole hand was covered using her or his own individual technique ("responsible application"). No specific rub-in instructions were given.

Based on the analysis of untreated skin areas in variations 2 – 11, three new rub-in techniques were developed with the aim of avoiding the most common gaps on hands. All three variations (14, 15 and 16) were performed with 3 mL, and consisted of 5 steps, two of which were identical:

Step 1: Palm to palm movement, backwards and forwards (twice).

Step 2: Rotational rubbing of closed fingertips in palm (twice). Repeat with other hand.

They differ in steps 3 to 5:

Variation 14

Step 3: The thumb of one hand is grasped in the palm of other with the fingers wrapping around the back. Rub thumb with palm (twice). Repeat with the other hand.

Step 4: The palm of one hand rubs the back of the other hand in a rotational movement up to the fingertips, then move closed hand back to the wrist (twice). Repeat with the other hand.

Step 5: The palm moves with spread fingers on back of the other hand up to the fingertips and back (twice). Repeat with the other hand.

Variation 15

Step 3: The palm of one hand rubs the back of the other hand in a rotational movement up to the fingertips, then back to the wrist. Repeat with the other hand.

Step 4: The thumb of one hand is grasped in the palm of the other hand and rotated twice. Repeat with other hand.

Step 5: The palm moves with spread fingers on the back of other hand backwards and forwards (twice). Repeat with the other hand.

Variation 16

Step 3: The thumb is placed in the palm of the other hand with its other fingers placed on the palm. Rub thumb with palm (twice). Repeat with other hand.

Step 4: The palm of one hand rubs the back of the other hand in a rotational movement up to the fingertips, then back to the wrist. (twice). Repeat with the other hand.

Step 5: The palm moves with spread fingers on back of the other hand up to the fingertips and back (twice). Repeat with the other hand.

### Assessment of untreated skin areas

An untreated skin area was defined as a gap of fluorescent dye on the hands irrespective of its location and size. Hands were evaluated under UV light using a Dermalux Box before the application of the hand rubs to make sure that no fluorescent dye was present before beginning the application, and after the application to determine the presence, size and location of untreated skin areas. The investigators were not aware of the type of treatment (blind study), except for the assessment of "responsible application" by the healthcare workers. A photograph was taken of each hand (palmar and dorsal side), after application. In addition, the location and size of the gaps were documented with a blue marker on a standard hand drawing. The size of untreated skin areas was determined by the investigator based on the drawings, and placed in one of four categories: 0%, up to 5%, 5% – 15%, and > 15%. For each type of application, the 15 drawings were scanned and super-imposed to gain a visual assessment of the locations with the highest proportion of untreated skin areas.

The analysis of data was done before the technique was revealed (unblinding). The proportion and size of untreated skin areas were presented descriptively.

## Results

**Figure 1:**
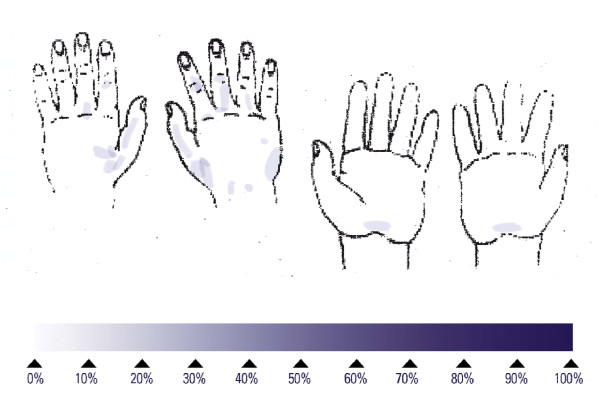
Superimposed untreated skin areas of 15 volunteers who performed the reference procedure for hygienic hand disinfection; median duration: 70 seconds.

**Figure 2:**
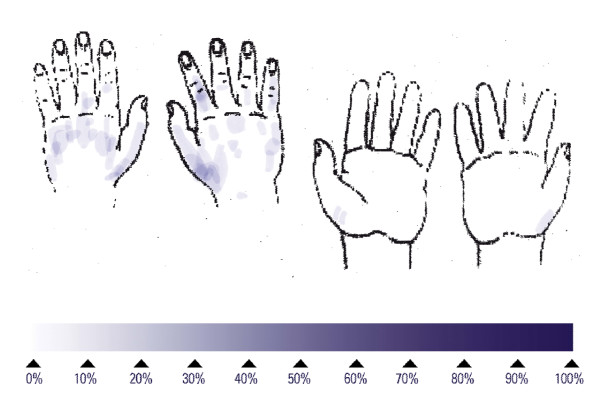
Superimposed untreated skin areas of 15 volunteers who performed each of the six rub-in steps for hygienic hand disinfection five times; median duration: 40 seconds.

**Figure 3:**
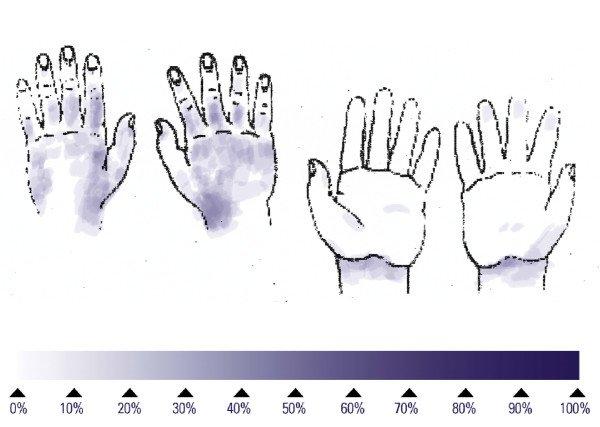
Superimposed untreated skin areas of 15 volunteers who performed each of the six rub-in steps for hygienic hand disinfection once; median duration: 17 seconds.

The reference procedure for hygienic hand disinfection with two applications of 3 mL lasted an average of 74.8 s and left 53% of subjects with at least one untreated area on the hands (Table 1), around the thumb or on the back of the hands (Figure 1). No large untreated areas were found (Table 1). Performing the hand disinfection with one aliquot of 3 mL, and 5 repetitions of the six steps, lasted an average of 37.3 s and left 67% of the subjects with at least one untreated area on the hands (Table 1), mainly on the back of the hands (Figure 2). No large untreated areas were found (Table 1). Reducing the number of repetitions of the six steps also reduced the mean duration to 16.7 s for one repetition. This short disinfection yielded at least one untreated area on the hands on all 15 subjects, mainly on the back of the hands (Figure 3). The proportion of large untreated areas was high at 53% (Table 1).

**Table 1:** Duration and quality of coverage of six techniques for hand disinfection (repetition of steps).

Type of hand rub	Repetitions of six steps (n)	Duration (median)	Duration (mean)	Location of untreated skin areas			Size of untreated skin areas (back of hands)			
				Whole hand	Palmar side	Dorsal side	0%	< 5%	5% – 15%	> 15%
PBHR*	1	17 s	16.7 s	100%	20%	100%	0%	7%	40%	53%
PBHR	2	23 s	24.7 s	93%	53%	87%	13%	40%	20%	27%
PBHR	3	25 s	25.7 s	93%	20%	93%	7%	67%	27%	0%
PBHR	4	35 s	34.9 s	87%	33%	80%	20%	53%	7%	20%
PBHR	5	40 s	37.3 s	67%	13%	67%	33%	53%	13%	0%
Reference alcohol	5 (twice)	70 s	74.8 s	53%	7%	53%	47%	47%	7%	0%

Application of a propanol-based hand rub in five treatment variations, or application of the reference alcohol: duration, and proportion and location of untreated skin areas, with size given for the back of hands; *PBHR = propanol-based hand rub.

**Figure 4:**
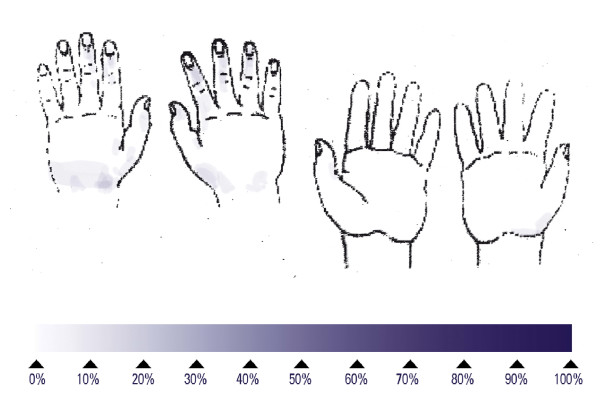
Superimposed untreated skin areas of 20 healthcare workers who performed the "responsible application" hygienic hand disinfection; median duration of 25.5 seconds.

Repeating the sequence of 5 steps lasted an average of 67.6 s and left 80% of the subjects with at least one untreated area on the hands (Table 2), mainly on the back of the hands. No large untreated areas were found (Table 2). Reducing the number of repetitions of the sequence also reduced the mean duration to 14.7 s for one repetition. This short disinfection time yielded at least one untreated area on the hands of 14 of the 15 subjects, mainly on the back. The proportion of large untreated areas was high at 27% (Table 2). When non-healthcare worker volunteers performed a "responsible application" hand disinfection, it lasted on average 25.0 s and left 53% of the subjects with at least one untreated area on the hands, mainly found on the back. No large untreated areas were found (Table 2). When healthcare workers performed a "responsible application" hand disinfection, it lasted an average of 27.8 s and left 55% of the subjects with at least one untreated area on the hands, again mainly on the back (Figure 4). No large untreated areas were found (Table 2).

**Table 2:** Duration and quality of coverage of seven techniques for hand disinfection (repetition of steps)

Type of treatment	Duration (median)	Duration (mean)	Location of untreated skin areas			Size of untreated skin areas (back of hands)			
			Whole hand	Palmar side	Dorsal side	0%	< 5%	5% – 15%	> 15%
1 repetition of sequence	14 s	14.7 s	93%	67%	93%	7%	33%	33%	27%
2 repetitions of sequence	28 s	26.7 s	80%	40%	80%	20%	33%	40%	7%
3 repetitions of sequence	37 s	37.4 s	73%	20%	73%	27%	40%	33%	0%
4 repetitions of sequence	48 s	49.7 s	80%	0%	80%	20%	47%	27%	7%
5 repetitions of sequence	73 s	67.6 s	80%	20%	80%	20%	60%	20%	0%
"responsible application" of non health-care worker test subjects	25 s	25.0 s	53%	13%	53%	47%	47%	7%	0%
"responsible application" of healthcare workers	25.5 s	27.8 s	55%	15%	55%	45%	45%	10%	0%

Comparison of hygienic hand disinfection when performed with a propanol-based hand rub in six treatment variations: duration, and proportion and location of untreated skin areas, with size given for the back of the hands.

**Figure 5:**
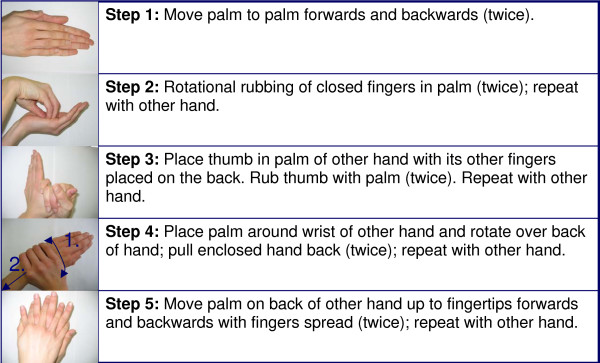
Description of a new rub-in technique (variation 14) which ensures a good coverage of hands within a clinically acceptable application time (median duration: 25 seconds).

**Figure 6:**
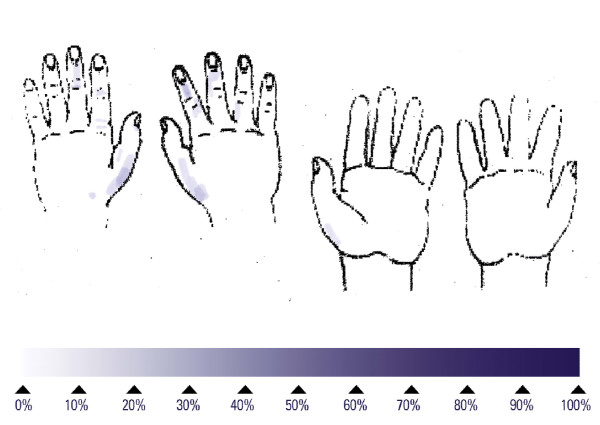
Superimposed untreated skin areas of 15 volunteers who performed a new rub-in technique for hygienic hand disinfection (variation 14); median duration of 25 seconds.

The three new rub-in techniques were all quite fast with an average duration between 22.3 s (variation 15) and 27.1 s (variation 16) (Table 3). Large untreated areas were not found with any of the new rub-in techniques. The lowest proportion of partially untreated hands was found with variation 14 (53%), followed by variation 16 (60%) and 15 (67%). Overall, rub-in technique 14 as described in Figure 5 yielded the best result with a short mean duration of 26.8 s and a high proportion of completely covered hands (47%). The few small untreated areas were distributed all over the back of the hand (Figure 6).

**Table 3:** Duration and quality of coverage of three new techniques for hand disinfection

Variation of rub-in technique	Duration (median)	Duration (mean)	Location of untreated skin areas			Size of untreated skin areas (back of hands)			
			Whole hand	Palmar side	Dorsal side	0%	< 5%	5% – 15%	> 15%
14	25 s	26.8 s	53%	7%	53%	47%	47%	7%	0%
15	23 s	22.3 s	67%	13%	67%	33%	60%	7%	0%
16	27 s	27.1 s	60%	7%	60%	40%	40%	20%	0%

Comparison of hygienic hand disinfection when performed with a propanol-based hand rub in three new treatment variations: duration, and proportion and location of untreated skin areas, with size given for the back of the hands.

## Discussion

We were able to show that the duration of the rub-in procedure is not the most relevant parameter to ensure a high quality of the hygienic hand disinfection procedure. Two good options were identified that ensure a high quality of hand coverage: allowing the individual to use his or her own "responsible application" procedure, (after having explicitly pointed out the importance of the complete coverage of both hands), or using a new five-step rub-in technique that ensures a better coverage of hands compared to the currently recommended six-step technique. Both options were found to be somewhat shorter than 30 s but greater than the insufficient 15 s. These findings lead to a recommendation of a 30 s application time for clinical practice.

It appears to be almost impossible to expect that a specific rub-in technique or "responsible application" can ensure that all or at least most hands are completely covered with the alcohol-based hand rub. In our study, the proportion of hands that were completely covered with the fluorescent hand rub ranged from 0% to 47%. However, most untreated skin areas were very small. Their location was mainly on the back of the hands but hardly ever on fingertips or palms. Fingertips and palms of the hands can be considered to have the highest clinical relevance, as they have the greatest chance of coming into direct contact with the patient or contaminated surfaces. Based on our rather small sample size, it appears appropriate to define a good quality hand disinfection as the absence of large untreated areas and the absence of untreated areas on fingertips and palms. This quality is not even guaranteed when the currently recommended rub-in procedure is followed in detail with one application of 3 mL and 5 repetitions of the six steps.

One limitation is that although 3 mL of hand rub were applied to the hands in all hand rub procedures, not all healthcare workers apply 3 mL to their hands in clinical practice. The applied volume of a hand rub may be lower than 3 mL depending on the type of dispenser, which may deliver considerably less than 3 mL if the lever is pushed only once. It has been shown recently that the application of 2.4 mL of a hand antiseptic is sufficient to cover most hands entirely [13]. Therefore, the results of the current study may not apply exactly to clinical situations in which a smaller amount of alcohol is used. If a smaller amount of alcohol is used in a clinical setting, the proportion of hands without untreated skin areas is likely to be even lower.

It is worth noting that the "responsible application" technique by both non-healthcare volunteers and healthcare workers provided an unexpectedly positive result. The "responsible application" technique among volunteers was done at the end of the second part of the study, consisting of variations 7 to 11. The positive outcome among non-healthcare volunteers can probably be explained by the repeated feedback given on their individual performance, which was provided immediately after each application. As this was done several times, the volunteers often knew the exact gaps in their personal treatment. When they were asked to perform the "responsible application" technique, they immediately made sure to cover the commonly missed parts of their hands. Therefore, individual teaching using a fluorescent hand rub appears to be helpful.

The healthcare workers were not given immediate feedback on untreated skin areas after performing the "responsible application", so these results reflect reality in clinical practice. The results were as positive as among non-professional volunteers with feedback. Although a study population of twenty healthcare workers does not allow general conclusions to be made, it appears that the healthcare workers' rub-in technique is not as poor as infection-control practitioners often claim. We also note that it may not be necessary to teach a specific rub-in technique with, for example, six steps, as long as healthcare workers know that they are responsible for covering their entire hands during hygienic hand disinfection. A specific rub-in technique with six steps may be too complex and difficult to memorize in clinical practice, so healthcare workers are likely to be ignorant about it. Since the specific rub-in technique with six steps also leads to a high proportion of untreated areas on the hands, the promotion of "responsible application" combined with individual training may be an appropriate way to ensure high quality in hand disinfection.

Quality in hygienic hand disinfection has various features. For many years, the overall compliance rate was considered to be the essential parameter for quality in hand disinfection, because it was shown that an increase of the overall compliance rate significantly reduced the rate of nosocomial infections [14]. Recently, however, observation of two other levels of compliance has been suggested: the specific compliance rate, to assess if the correct procedure was performed (e.g. a hand disinfection when it was indicated, or a hand wash when it was indicated); and the correct performance of the indicated hand hygiene procedure [15]. Currently, it is not known if an improved rub-in technique for hygienic hand disinfection can reduce the rate of nosocomial infections. It could, however, be a simple step to improve the quality of the hand disinfection procedure itself.

For other parameters in hand disinfection, such as the efficacy of the hand rub [1], the personal perception of the hand rub [16], or availability and functionality of a dispenser [17,18], the potential to reduce the rate of nosocomial infections is unclear. Nonetheless, they are considered to be essential for optimum quality of hygienic hand disinfection in clinical practice. The overall application time in hygienic hand disinfection does not seem to have a major impact, however, because 25 to 30 s is required for the evaporation of the routinely applied 3 mL of alcohol [19], an average application time that is also described as necessary to cover both hands in the upcoming WHO guidelines on hand hygiene [20]. Hand washing requires even more time [21,22]. Even if a hand-rub formulation is effective within 15 s, the hands are unlikely to be dry after 15 s, if 3 mL are applied. Thus, a reduction of the application time to 15 or 20 s has no real clinical relevance, as it does not change the practical application of the hand rub. If the price of a quick rub-in procedure of 15 s is the occurrence of large untreated areas as shown in our study, an application time of 15 s certainly has an overall negative benefit-risk balance.

## Conclusion

The clinical practice of hand disinfection is apparently better than infection control-practitioners often anticipate. Based on our data, a high-quality hygienic hand disinfection is not possible within 15 s. A 30 s application time can, however, be recommended for clinical practice. The currently recommended six steps of EN 1500 are not really suitable for use in clinical practice because they yield poor results. "Responsible application" or the new technique described here is recommended.

## Competing interests

The first four authors are paid employees of Bode Chemie GmbH & Co. KG, Hamburg, Germany.

## Authors' contributions

All authors designed the study, GK analysed the data and wrote the manuscript, all authors read and approved the final manuscript.

## Pre-publication history

The pre-publication history for this paper can be accessed here:


